# Protocol for the cost-consequence and equity impact analyses of a cluster randomised controlled trial comparing three variants of a nutrition-sensitive agricultural extension intervention to improve maternal and child dietary diversity and nutritional status in rural Odisha, India (UPAVAN trial)

**DOI:** 10.1186/s13063-019-3388-2

**Published:** 2019-05-27

**Authors:** Hassan Haghparast-Bidgoli, Jolene Skordis, Helen Harris-Fry, Sneha Krishnan, Meghan O’Hearn, Abhinav Kumar, Ronali Pradhan, Naba Kishore Mishra, Avinash Upadhyay, Shibananth Pradhan, Amit Kumar Ojha, Sarah Cunningham, Shibanand Rath, Tom Palmer, Peggy Koniz-Booher, Suneetha Kadiyala

**Affiliations:** 1 University College London, Institute for Global Health, 30 Guilford Street, London, WC1N 1EH UK; 2 London School of Hygiene and Tropical Medicine, Keppel Street, London, WC1E 7HT, UK; 3 Tufts University, Friedman School of Nutrition Science and Policy, 150 Harrison Avenue, Boston, MA 02111 USA; 4 Digital Green, S-26 to 28, 3rd Floor, Green Park Extension Market, New Delhi, 110016 India; 5 VARRAT (Voluntary Association for Rural Reconstruction and Appropriate Technology), Boulakani Baradang, Mahakalpara Kendrapad, Odisha 754224 India; 6 Ekjut, 556 B-Ward No 17-Potka, Chakradharpur, Jharkhand 833102 India; 7 Harvard T.H. Chan School of Public Health, 677 Huntington Avenue, Boston, MA 02115, USA; 8 Strengthening Partnerships, Results, and Innovations in Nutrition Globally, JSI Research and Training Institute, Inc., 1616 Fort Myer Drive 16th Floor, Arlington, VA 22209 USA

**Keywords:** Cost-consequence analysis, Nutrition-sensitive agriculture, Participatory learning and action, Women’s groups, Dietary diversity, Maternal and child nutrition, India

## Abstract

**Background:**

Undernutrition causes around 3.1 million child deaths annually, around 45% of all child deaths. India has one of the highest proportions of maternal and child undernutrition globally. To accelerate reductions in undernutrition, nutrition-specific interventions need to be coupled with nutrition-sensitive programmes that tackle the underlying causes of undernutrition. This paper describes the planned economic evaluation of the UPAVAN trial, a four-arm, cluster randomised controlled trial that tests the nutritional and agricultural impacts of an innovative agriculture extension platform of women’s groups viewing videos on nutrition-sensitive agriculture practices, coupled with a nutrition-specific behaviour-change intervention of videos on nutrition, and a participatory learning and action approach.

**Methods:**

The economic evaluation of the UPAVAN interventions will be conducted from a societal perspective, taking into account all costs incurred by the implementing agency (programme costs), community and health care providers, and participants and their households, and all measurable outcomes associated with the interventions. All direct and indirect costs, including time costs and donated goods, will be estimated. The economic evaluation will take the form of a cost-consequence analysis, comparing incremental costs and incremental changes in the outcomes of the interventions, compared with the status quo. Robustness of the results will be assessed through a series of sensitivity analyses. In addition, an analysis of the equity impact of the interventions will be conducted.

**Discussion:**

Evidence on the cost and cost-effectiveness of nutrition-sensitive agriculture interventions is scarce. This limits understanding of the costs of rolling out or scaling up programs. The findings of this economic evaluation will provide useful information for different multisectoral stakeholders involved in the planning and implementation of nutrition-sensitive agriculture programmes.

**Trial registration:**

ISRCTN65922679. Registered on 21 December 2016

**Electronic supplementary material:**

The online version of this article (10.1186/s13063-019-3388-2) contains supplementary material, which is available to authorized users.

## Background

Maternal and child undernutrition are major global challenges with substantial consequences for health, human development and economic productivity. Undernutrition causes around 3.1 million child deaths annually, approximately 45% of all child deaths [1]. Maternal and child undernutrition also have substantial short-term and life-long adverse consequences including adverse pregnancy outcomes, reduced child survival, curtailed cognitive and educational performance, and increased incidence of chronic disease in adulthood [2, 3].

The economic costs of undernutrition are substantial, disproportionately affecting the most vulnerable in low- and middle-income countries (LMICs) [4, 5]. The economic productivity loss to individuals caused by undernutrition is estimated at more than 10% of lifetime earnings, while losses to national economies are estimated to be around 2–3% of Gross Domestic Product [6].

While India has made progress in reducing maternal and child undernutrition, the prevalence of child undernutrition remains extremely high, with 38% of children under 5 years of age being stunted, 21% wasted and 58% anaemic [7]. The poorest households and Scheduled Tribe (ST) and Scheduled Caste (SC) communities are disproportionately affected [8, 9]. In addition, it is estimated that almost a quarter of women aged 15–49 years in India are underweight and over half are anaemic [9].

There is strong evidence that nutrition-specific interventions, i.e. interventions that address immediate determinants of undernutrition, could improve nutrition and health outcomes if implemented at a national scale in countries with the highest burden of undernutrition [2], such as India. However, to accelerate reductions in undernutrition, nutrition-specific interventions need to be linked with nutrition-sensitive programs that tackle the underlying causes of undernutrition in other sectors, such as agriculture [10]. Emerging evidence shows positive effects of nutrition-sensitive agriculture (NSA) interventions on nutrition outcomes, such as maternal dietary diversity, micronutrient intake, child wasting and maternal underweight, and reductions in anaemia among mothers and children [11]. Potential explanations for these effects include increased agriculture production, increased household income, a reduction in women’s workload and an increase in women’s decision-making power [10, 12]. However, there remains a paucity of evidence on the impact of these interventions on maternal nutrition outcomes, as well as their cost-effectiveness.

### Upscaling Participatory Action and Videos for Agriculture and Nutrition (UPAVAN) trial

The UPAVAN trial is a four-arm, cluster randomised controlled trial, implemented in Odisha, India. The trial aims to test the nutritional and agricultural impacts of: (1) a participatory agriculture extension platform of women’s groups viewing videos about NSA practices, and follow-up home visits to encourage the adoption of the practices presented in the videos; (2) a modified version of NSA intervention that incorporates videos on nutrition-specific behaviour change and (3) another modified version integrating a participatory learning and action (PLA) approach of collective identification, prioritisation, and community action to solve local problems. In addition to these three interventions, health system strengthening (HSS) activities, including 2 days of training in maternal, infant and young child nutrition for frontline nutrition and health workers, will be provided in all study areas, including the control arm.

A detailed description of the UPAVAN trial and its interventions are presented in the trial protocol [13]. This paper aims to describe the methodology for the economic evaluation of the trial.

### Cost-effectiveness evidence of NSA interventions and maternal and child undernutrition

While there is some evidence for the positive impact of NSA programmes on maternal and child undernutrition [11], evidence on their cost-effectiveness is scarce. This constrains any investment case and limits our understanding of the feasibility of scaling up these interventions [10, 11]. Among those studies reporting economic evidence, nearly all are modelling exercises and the majority focus on biofortification programs [14, 15], with only one focussed on home garden programs [16]. Furthermore, although participatory interventions with women’s groups using a PLA approach have been shown to be highly cost-effective in improving maternal and newborn health [17], the evidence of their effectiveness and cost-effectiveness for improving dietary intake [18, 19] and nutrition outcomes [20, 21] is mixed. There is also no evidence, or very limited evidence, on the cost or cost-effectiveness of multisectoral nutrition-sensitive interventions, beyond biofortification and home gardens, in particular using digital platforms. The only economic evidence available for the use of digital platforms is the costing study conducted by Khurana et al. [22], reporting the cost of piloting one of the nutrition-specific intervention components of the UPAVAN intervention.

One of the main reasons for the scarcity of cost and cost-effectiveness evidence on NSA interventions in general, might be related to their multisectoral nature. This makes collecting and interpreting cost (and outcome) data complex, time-consuming and expensive [11]. Moreover, cost-effectiveness evaluations of NSA interventions face another challenge that stems from their multisectoral nature. These analyses focus only on the health outcome either using composite measures of mortality and morbidity, such as Disability Adjusted Life Years (DALYs) or natural units of outcome, such as cases of stunting averted. These measures do not capture the full range of benefits and outcomes, including non-health benefits, generated by NSA interventions; such as food security, women’s empowerment, knowledge and increased income from agriculture production among others [23].

The economic evaluation of the UPAVAN interventions will thus take the form of a cost-consequence analysis (CCA). This approach, to some extent, can address the challenges mentioned above. In CCA, all relevant costs and outcomes of the interventions are listed, in a tabular format, without aggregating into ratios, allowing policymakers to compare the incremental costs with the incremental consequences of the different interventions. CCA is recommended for complex multisectoral public health interventions, such as NSA interventions, that have multiple health and non-health effects, which may be difficult to measure in a common unit [24, 25]. Considering that these interventions are relevant to a wide range of stakeholders, using this approach allows different stakeholders to differentiate where costs and benefits might be accrued in their own sector, e.g. health care, agriculture, and social welfare.

### Aim and objectives

The economic evaluation of the UPAVAN trial will consist of a cost-consequence analysis of the three UPAVAN interventions from a societal perspective.

The specific objectives of the UPAVAN economic evaluation are:

1. To estimate the costs of designing and implementing the UPAVAN interventions (programme costs)
2. To calculate the costs to the health care system and other sectors, of increased care-seeking and demand for their services, as a result of the intervention
3. To measure changes in household care-seeking costs and any costs of adopting new practices as a result of the interventions
4. To present the mean and incremental costs and outcomes of the interventions as a cost-consequence analysis, compared with the status quo

In addition to the above objectives, an equity impact analysis of the UPAVAN interventions will be carried out to evaluate how costs and consequences of the interventions are distributed among different sub-groups within the target population.

## Methods

### Study setting and participants

The UPAVAN trial is set in four administrative blocks (Patna, Keonjhar Sadar, Harichandanpur and Ghatgaon blocks) in Keonjhar district, Odisha. The total population of Keonjhar district is estimated at 1.8 million, which is 86% rural and agrarian [26]. Keonjhar is a tribal district with around 57% of its population comprised of SCs and STs (12% and 45%, respectively) [26]. It has one of the highest undernutrition rates among women and children in India. The prevalence of underweight and anaemia among women in Keonjhar is 30% and 40%, respectively [9]. Approximately 45% of children aged under 5 years are stunted and 19% are wasted [9]. Among children of 6–23 months of age, less than 10% are fed a minimum acceptable diet [9].

All women aged 15 to 49 years residing permanently (i.e. lived there for more than 6 months) in the 148 trial clusters are eligible to participate in the interventions (i.e. ‘intervention participants’). The trial will evaluate impact on outcomes in ‘trial participants’, i.e. children under 2 years of age, their mothers or female primary caregivers, and their households. The UPAVAN trial will evaluate impacts on outcomes for ‘trial participants’ through baseline and endline repeat cross-sectional surveys.

### Study design

One hundred and forty-eight clusters were randomly allocated to the four trial arms to give 37 clusters per arm. A cluster is defined as one or two villages and surrounding hamlets, with a minimum population size of 800 people. Three arms will receive different packages of intervention activities, while the fourth arm acts as a control. In all four arms, basic health system strengthening activities will be implemented. The trial will run from December 2015 to May 2020 (53 months), which includes 15 months of trial set-up and intervention development and 32 months of intervention implementation, starting from April 2017 to October 2019.

### Description of the interventions and comparator

Three UPAVAN interventions build on Digital Green’s participatory video-based approach [27]. Building on existing community institutions and public systems, the Digital Green approach is a method of delivering agriculture extension using low-cost participatory videos with women’s and farmers’ groups. The approach comprises four steps:

1. Content of videos (or agriculture practices to be promoted) is identified through participatory consultation with community stakeholders
2. Community members are identified and trained on video production and editing. Actors (who are generally early adopters of the practices or an agriculture extension worker, if a new concept is to be introduced to the community) for the videos are identified
3. Videos are disseminated through community groups, such as self-help groups, by a local trained facilitator (called a community support person or CSP). Facilitators conduct follow-up visits to the viewers, after each dissemination session, to verify whether they adopted the practices and/or can recall the key messages in the videos
4. Detailed process data on videos, such as coverage or group attendance, knowledge recall and adoption of practices during follow-up visits are recorded in a customised data monitoring system. The process data, plus qualitative feedback collected during dissemination meetings and follow-up visits by the facilitators, help to identify content for future videos

#### UPAVAN interventions

**Fig. 1:**
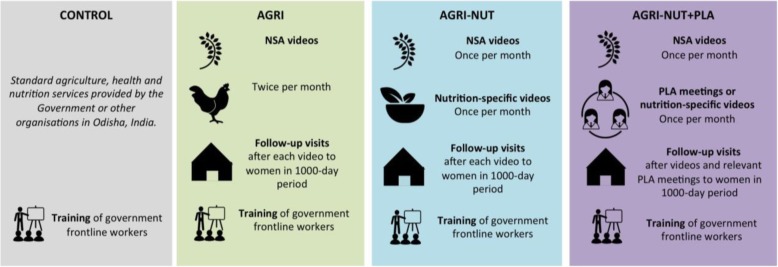
Outline of UPAVAN for each intervention arm and control or comparator [13]

Based on a feasibility study [28] and formative research [29], some key innovations were added to the Digital Green foundational approach for the UPAVAN trial: (1) made videos topics or practices promoted ‘nutrition-sensitive’, (2) coupled nutrition-specific video topics with NSA topics and (3) incorporated nutrition-specific PLA approaches with the NSA extension intervention, to encourage uptake of nutrition-specific behaviours. An overview of the UPAVAN interventions and control/comparator is presented in Fig. 1, accompanied with a brief description of the interventions below. A full description of the UPAVAN interventions is presented elsewhere [13].

The UPAVAN interventions are implemented by VARRAT, a non-governmental organisation in Odisha state, and a number of other partner organisations, i.e. Digital Green, Ekjut, John Snow Inc. Research and Training Institute (JSI RTI) and the London School of Hygiene and Tropical Medicine (LSHTM), provide technical assistance for development and implementation of the interventions.

##### AGRI

In the nutrition-sensitive agricultural extension intervention (AGRI) intervention arm, women’s groups view and discuss two NSA videos each month for 32 months. After each video viewing, follow-up visits are made to all participating pregnant women and mothers of children aged 0–23 months (primary beneficiaries of the trial). These follow-up visits aim to: check whether the participants can recall the key messages in the videos or have adopted the key practices, reinforce the messages shown in the videos, and encourage participants to attend next meetings. Another purpose of the visits is to strengthen the link between participants and community frontline workers, and to refer visibly ill or malnourished children to community workers or other health providers. CSPs run the video dissemination meetings and conduct the home visits.

##### AGRI-NUT

In the nutrition-sensitive agricultural extension and nutrition-specific behaviour-change intervention (AGRI-NUT) intervention arm, women’s groups view and discuss two videos each month, one video covering NSA topics and one covering nutrition-specific topics, accompanied with follow-up visits as outlined above.

##### AGRI-NUT+PLA

In the nutrition-sensitive agricultural extension and nutrition-specific behaviour-change intervention with a Participatory Learning and Action approach (AGRI-NUT+PLA) intervention, each month women’s groups view and discuss one video on NSA and have one PLA meeting focussed on nutrition-specific behaviour-change. PLA meetings are either discussion-based meetings or facilitated disseminations of videos on nutrition-specific topics.

In contrast with AGRI-NUT arm, which follows Digital Green’s foundational approach of video content identification, production of nutrition-specific videos in this arm results from discussions in PLA meetings. Also, in this arm, follow-up visits are conducted only when relevant (i.e. only for NSA videos or PLA meetings that promote clear nutrition-specific messages).

The PLA cycle consists of four phases, which guide the groups to identify and prioritise locally important maternal and child nutrition problems (phase 1), explore the causes and consequences of the prioritised problems and decide on locally feasible strategies (phase 2), then implement (phase 3) and evaluate (phase 4) these strategies.

##### Control or comparator

The control arm or comparator receives standard government services or services provided by non-governmental organisations. Moreover, in both intervention and control arms, activities are undertaken to strengthen the capacity of government frontline nutrition and health workers (Anganwadi workers (AWWs) and accredited social health activists (ASHAs)). Frontline health and nutrition workers will receive 2 days of training in maternal, infant and young child nutrition.

### Measurement of outcomes/effectiveness

The outcomes of the interventions will be assessed through repeat cross-sectional household surveys at baseline (2016–2017) and endline (2019–2020). The outcomes of the interventions will be assessed relative to the control arm and not relative to each other. Analysis of the trial outcomes will be carried out on groups as randomised (i.e. intention-to-treat) at the individual level, adjusted for clustering. Since the trial outcomes will be evaluated using repeat cross-sectional surveys, loss-to-follow-up of specific individuals will not be an issue. Moreover, survey completion rates are expected to be high in this context, so any participants with missing data will be excluded from primary outcome analysis [13].

### Primary outcome

The UPAVAN trial has two primary outcomes: (1) child dietary diversity, measured as proportion of children 6–23 months of age consuming four or more out of seven food groups per day, based on a 24-h dietary recall, and (2) mean maternal body mass index (kg/m^2^) of non-pregnant, non-postpartum mothers or female primary caregivers of children 0–23 months of age.

### Secondary outcomes

The secondary outcomes of the trial are: (1) maternal dietary diversity, measured as proportion of mothers or female primary caregivers consuming five or more out of 10 food groups per day, based on a 24-h dietary recall and (2) child wasting, measured as proportion of children with a weight-for-height z-score < − 2 standard deviations (SD).

In addition to the primary and secondary outcomes, the trial has a number of other health and non-health outcomes including maternal and child low mid-upper arm circumference, maternal and child haemoglobin (Hb) concentrations, infant and young child feeding practices, women’s decision-making, women’s time use, gender parity, household economic status and food security and household agricultural production. A complete list of the trial outcomes and their corresponding indicators is listed in Table 2 of the trial protocol [13].

### Identification, measurement and valuation of resource use

The economic evaluation of the UPAVAN interventions will be in the form of a cost-consequence analysis, conducted from a societal perspective [30, 31]. This includes measuring all costs incurred by the implementing agency (programme-provider costs), community frontline workers and other health care providers, and users, i.e. intervention participants and their households. All associated and measurable outcomes or benefits associated with the intervention over the intervention time horizon will also be enumerated.

Table 1 provides an overview of the resource use and cost measures used. The proposed methods for measuring programme costs, community frontline workers and other health care providers’ costs and participants/household costs are described in more detail in the following sections.

**Table 1:** Overview of resource use and costs measures included in the economic evaluation of the UPAVAN interventions

Perspective/cost category		Type of costs	Description	Sources of data	Sample size
Provider	Program	Direct	Costs of designing and implementing the interventions	1. Project accounts of the implementing agencies 2. Interviews with the project staff	N/A
		Indirect	Opportunity cost of donated items, volunteer times	1. Interviews with the project staff 2. Project records on volunteer involvement 3. Project accounts	N/A
	Health (and non-health) providers	Direct	Changes in demand/utilisation of nutrition and health services from community health workers (CHWs) and other health providers in the study area	1. Endline evaluation survey (for information on changes in health-seeking behavior) 2. Published data on unit costs of services	All participants from the endline evaluation survey (around 4700 households)
		Indirect	The opportunity cost of the time spent by the community providers participating in the interventions	1. Project records on frontline workers’ participation in intervention 2. Project records on numbers of health service strengthening meetings and attendants 3. Published reports on frontline workers’ salary and local minimum wage information	N/A
Participants/households		Direct	1. Household expenditure on food and non-food 2. Costs of health-seeking for the participants and their households 3. Costs of adopting the practices	1. Household consumption expenditure survey at endline 2. Endline evaluation survey 3. (a) Monitoring system – practice adoption reports collected by CSPs during follow-up visits ᅟ(b) Endline evaluation survey ᅟ(c) Qualitative interviews with a sub-sample of participants ᅟ(d) Interviews with the project staff	1. A random sample of 50% of endline participants (around 2350 households 2. All participants from the endline evaluation survey (~ 4700 households) 3. (a) All adoption records from follow-up visits: ~ 550 women × 54 planned meetings ᅟ(b) All individual attended the group meetings from the endline evaluation survey ᅟ(c) To be decided ᅟ(d) N/A
		Indirect	1. Opportunity cost of participation in the group meetings and home visits 2. Opportunity cost of adopting practices	1.Endline evaluation survey 2.Time-use surveys 3.Published reports with local minimum wage information 4. Qualitative interviews with a sub-sample of participants	1. All individual attended the group meetings at the endline evaluation survey 2. All women (~ 4700) and 50% of men (~ 2350) at endline 3. N/A 4. To be decided

*N/A* not applicable

### Costs to programme providers

Programme costs are those incurred by the programme provider, i.e. VARRAT, and other partner organisations mentioned earlier. A combination of different costing approaches including activity-based costing [32], expenditure approach and ingredients approach [33] will be employed to estimate direct (financial) and indirect (opportunity) costs of designing and implementing the UPAVAN interventions. In brief, main intervention components and major activities under each component will be identified and defined as cost centres and the costs (mainly extracted through the expenditure records or estimated in case of opportunity costs) will be allocated to these cost centres.

#### Financial or expenditure data

Financial or accounting costs comprise the majority of the programme costs. These costs will be collected prospectively from the project accounts or expenditure records of the implementing and technical partners and entered into a programme costing tool in Microsoft Excel on an annual basis. The tool is divided into different sections based on line items, including staff, materials, capital, and joint costs and intervention components (or cost centres), i.e. AGRI, NUT, PLA, health system strengthening and monitoring and evaluation (or quality assurance and monitoring information system). Joint costs are administration, overhead and other costs that shared by several intervention components. These costs will be allocated between the programme components using key informant interviews with the project leads and time use data from regular staff timesheets. All costs related to the research activities will be excluded from the analysis. However, the research team inputs into the interventions’ design are captured under start-up costs.

#### Donated items and opportunity costs

Project accounts and expenditure reports do not routinely capture resources, such as donated items and volunteer time. These resources need to be identified and converted to economic costs using their current market value [33–35]. Common donated resources are equipment or other capital items owned or donated by the implementing agency. These include items purchased by previous projects but used in UPAVAN. The donated resources will be identified through key informant interviews with the project staff.

Project volunteers are mainly community members who are involved in video production; for example, the actors in the videos. Detailed information on the number of community volunteers and time spent will be documented by the project. The opportunity cost of the time invested by the volunteers will be estimated using published local wage rates (https://paycheck.in/salary/minimumwages/orissa).

### Costs to public health care providers

UPAVAN interventions may increase demand for services provided by community frontline health and nutrition workers, i.e. AWW, ASHA and auxiliary nurse midwife (ANM), and other health care providers, such as malnutrition treatment centres or nutrition rehabilitation centres and primary health centres in the project area. In addition, frontline workers and other providers will have incurred a time cost of their direct involvement in the production of videos, attending women groups and attending health systems strengthening activities (Table 1).

#### Cost of changes in utilisation of health care providers

Changes in the utilisation of health and nutrition services provided by the frontline workers and other health care providers will be collected from study participants in both intervention and control clusters at the UPAVAN endline evaluation survey. Changes in service use between intervention and control areas will be attributed to the UPAVAN interventions. Moreover, any changes in use of services provided by public providers other than those mentioned above will be identified during the endline evaluation survey. Costs of changes in service use will be estimated using published data on the unit costs of related health and nutrition services.

#### Opportunity cost for front line workers

Frontline workers and agriculture extension workers are involved in the production of some videos and attend a number of the women groups. Moreover, health systems strengthening activities include 2 days of training in maternal, infant and young child nutrition for the frontline workers in the intervention and control arms. Detailed information on the involvement of frontline workers and extended agriculture workers is being documented by the project. The opportunity cost of involvement in the interventions, or the value of the time spent by frontline workers will be measured as a proportion of their salary, using publicly available data on their salaries. However, it should be acknowledged that the involvement in the interventions might provide some long-term benefits to the frontline workers; for example, in terms of improving quality of their work or lightening their workload. Although the perceived benefits of the interventions will be explored qualitatively but they would not be quantified in this study.

### Costs to participants and their households

UPAVAN interventions may affect the expenditure of study participants and their households in a number of ways. Impact on participants and their families may include changes in care-seeking behaviour and associated costs, and changes in household food and non-food consumption patterns and spending (mainly due to direct and indirect costs of adopting agriculture or nutrition-promotion practices). It also includes the time cost of participation in group meetings, follow-up home visits, and the actions taken by the groups. The interventions and behaviours promoted may also affect the allocation of time within a household.

#### Health care-seeking costs

UPAVAN interventions may increase participants’ seeking advice and care for child malnutrition and other related illnesses, such as diarrhoea, fever, cough, and maternal care, such as antenatal and postnatal care. This advice may be sought from both formal and informal providers. Information on the costs of care-seeking will be collected from all the primary caregivers of children under 2 years old, at the endline evaluation surveys. The difference in health care seeking costs for the participants (and their households) will be estimated and compared between intervention and control areas.

#### Changes in household expenditure

Households’ food and non-food expenditure are captured in a household consumption and expenditure survey at the end-line on a random sub-sample of around 2350 households. Differences in household expenditure including total spending, food spending, non-food spending and per-capita spending over the intervention period will be compared between interventions and control areas.

#### Opportunity cost of participation in the interventions

Attending the group meetings and follow-up home visits incurs direct and indirect costs to participants and their family. These costs include the cost of getting to the group and the time cost of participation in the group meetings, i.e. travel time and time spent in the group, follow-up home visits, adopting agriculture or nutrition-specific practices, or participating in the actions taken by the group.

Information on the potential direct and time costs of participation in the group will be collected from the individuals attended the group meetings at the endline evaluation survey. The interventions promote a number of agriculture or nutrition-specific practices which might cause both direct and indirect costs to the participants. The interventions’ monitoring information system records detailed information on attendance and frequency of the group meetings and individual-level data on adoption of self-reported behaviours. However, it will be challenging to estimate the direct (and indirect) cost of all the practices adopted due to high cost of collecting more detailed information on additional time and inputs required for each practice. We will explore collecting these data through qualitative surveys with a sample of the participants in the intervention arms, complemented with key informant interviews with the project staff about the inputs required for the promoted practices. However, it might not possible to fully identify and measure the indirect costs of adopting the practices; for example, costs of labour time savings or labour substitution.

The actions promoted by the interventions may also affect the allocation of time within households. Changes in the time allocation within households will be collected with a time use survey on all women (around 4700) and a sub-sample of men (50%, around 2350) in the interventions and control areas, as part of both the baseline and endline evaluation survey.

### Analyses

As mentioned earlier, the economic evaluation of the UPAVAN interventions will take the form of a cost-consequence analysis, tabulating all relevant costs and outcomes of the interventions without aggregating them into a ratio. An incremental analysis of the costs and consequences of the alternative interventions will be conducted; comparing additional costs and outcomes generated by AGRI, AGRI-NUT and AGRI-NUT+PLA with the control or status quo. In addition, the costs will be disaggregated by programme provider, health care providers and participants.

All estimated costs will be presented in 2019 prices in both Indian Rupees and International Dollars (INT$), adjusted for inflation using the Indian Consumer Price Index and converted to 2019 INT$ using the Purchasing Power Parity conversion factor for India. Moreover, the costs will be discounted at 3%, the standard discount rate recommended by WHO-CHOICE [36] and the Gates/iDSI Reference Case for Economic Evaluation [37]. A series of univariate and multivariate sensitivity analyses will assess effect of the main cost drivers on the results. The study design, analytical methods and reporting of the findings will follow the Consolidated Health Economic Evaluation Reporting Standards (CHEERS) Statement [38].

The possibility of conducting a cost-benefit analysis and modeling state-wide and national scale up of the UPAVAN interventions will be explored.

### Equity impact of the UPAVAN interventions

An equity impact analysis of the interventions will be also carried out to evaluate how costs and consequences of the interventions are distributed among different sub-groups within the target population. This will be done through sub-group analyses of all trial outcomes and individual-level costs (e.g. cost of seeking care, opportunity costs of involving in the intervention, etc.) based on selected socio-economic dimensions of the target population, such as education, household wealth and tribal and caste status.

## Discussion and conclusions

There is a lack of evidence on the cost and cost-effectiveness of NSA interventions, which limits the assessment of their potential scale-up feasibility. The current study will contribute to the scarce evidence in this area and will be the first economic evaluation of a participatory, NSA extension platform coupled with a nutrition-specific behaviour-change intervention, and PLA approach.

The study adopts a cost-consequence analysis approach which is suitable for multisectoral interventions, such as NSA interventions, making it possible to report all health and non-health impacts of interventions, as well as incorporating the equity impact of the interventions. This approach is expected to be desirable to different stakeholders in the planning and implementation of such programmes, facilitating informed multi-criteria decision-making. Although this approach, by including all health and non-health outcomes, addresses one of the main limitations of the cost-effectiveness of NSA interventions, research needs to be conducted in identifying or developing composite outcome measures that capture multiple benefits of such interventions.

The UPAVAN trial is not powered to test the differences between the interventions as this required a large sample size and as a result more resources; however, the possibility of a direct comparison between the three interventions will be explored. Moreover, we anticipate that the estimating of the direct and indirect costs of the practices adopted, both at individual or community levels, will be challenging. We will explore collecting required data through qualitative surveys and key informant interviews with the project staff. However, it might not possible to fully identify and measure the indirect costs of adopting the practices.

### Trial timeline and status

**Fig. 2:**
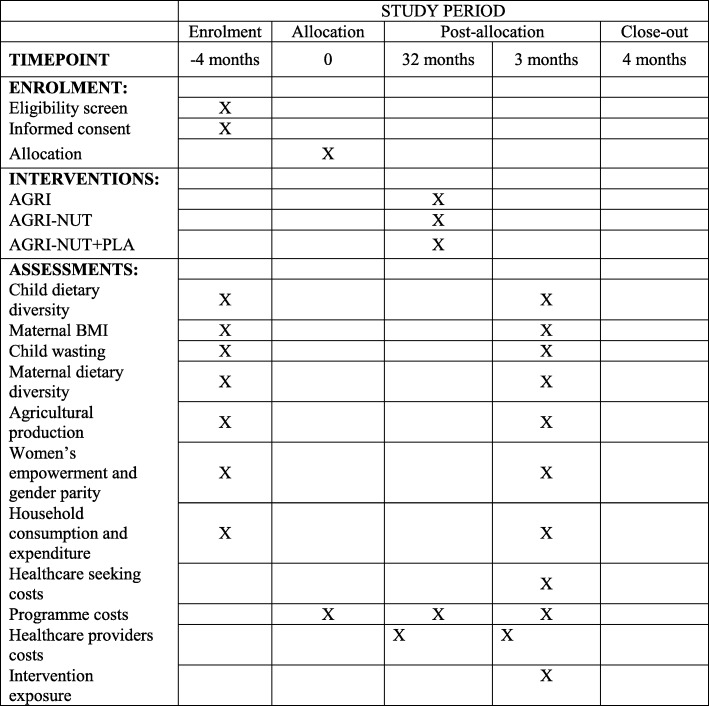
Standard Protocol Items: Recommendations for Interventional Trials (SPIRIT) Figure illustrating the schedule of enrolment, interventions and assessments

This is protocol version number 1, finalised on 30 November 2018. The intervention implementation started in March 2017 and will end in October 2019 (32 months of intervention exposure). Endline impact evaluation survey will start in November 2019 and continue until May 2020. The detailed timing of data collection points, based on the Standard Protocol Items: Recommendations for Interventional Trials (SPIRIT) timeline, can be seen in Fig. 2. The SPIRIT Checklist is also available as Additional file 1.

## Additional file

Additional file 1: Standard Protocol Items: Recommendations for Interventional Trials (SPIRIT) 2013 Checklist: recommended items to address in a clinical trial protocol and related documents*. (DOC 122 kb)

## Abbreviations

AGRI: Nutrition-sensitive agricultural extension intervention
AGRI-NUT: Nutrition-sensitive agricultural extension and nutrition-specific behaviour-change intervention
AGRI-NUT+PLA: Nutrition-sensitive agricultural extension and nutrition-specific behaviour-change intervention with a Participatory Learning and Action approach
ANW: Auxiliary nurse midwife
ASHA: Accredited social health activist
AWW: Anganwadi worker (AWW)
BMI: Body mass index
CCA: Cost consequence analysis
CHEERS: Consolidated Health Economic Evaluation Reporting Standards
CSPs: Community support persons
FLWs: Front-line workers
HSS: Health systems strengthening
INT$: International dollars
LMICs: Low- and middle-income countries
MUAC: Mid-upper arm circumference
NSA: Nutrition-sensitive agriculture
PHC: Primary health centres
PLA: Participatory learning and action
SC: Scheduled Caste
ST: Scheduled Tribe
UPAVAN: Upscaling Participatory Action and Videos for Agriculture and Nutrition
